# Workflow optimization for syndromic diarrhea diagnosis using the molecular Seegene Allplex™ GI-Bacteria(I) assay

**DOI:** 10.1007/s10096-020-03837-4

**Published:** 2020-02-06

**Authors:** Stefan Zimmermann, Susanne Horner, Martin Altwegg, Alexander H. Dalpke

**Affiliations:** 1 Department for Infectious Diseases, Medical Microbiology and Hygiene, University Hospital Heidelberg, Heidelberg, Germany; 2 Bioanalytica AG, Lucerne, Switzerland; 3 Institute of Medical Microbiology and Hygiene, Medical Faculty, Technische Universität Dresden, Dresden, Germany, Fetscherstraße 74, 01307 Dresden, Germany

**Keywords:** Syndromic panels, Diagnostics, Bacterial diarrhea, Clinical microbiology, Molecular, Assay

## Abstract

Syndromic panel-based molecular testing has been suggested to improve and accelerate microbiological diagnosis. We aimed to analyze workflow improvements when using the multiplex Seegene Allplex™ GI-Bacteria(I) assay as a first-line assay for bacterial diarrhea. Technical assay evaluation was done using spiked stool samples and stored patient samples. After implementation of the assay in the routine clinical workflow, an analysis of 5032 clinical samples analyzed by the Seegene assay and 4173 control samples examined by culture in a similar time period 1 year earlier was performed. Sensitivity of the assay was shown to be between 0.4 and 95.9 genome equivalents/PCR. For 159 positive patient samples with a composite reference of culture and/or a molecular assay, the sensitivity of the assay was 100% for *Campylobacter*, 92% for *Salmonella*, 89% for *Aeromonas*, and 83% for *Shigella*. Sensitivity for *C. difficile* toxin B detection was 93.9%. The comparison of clinical samples obtained in two 8-month periods showed increased detection rates for *Aeromonas* (2.90%vs. 0.34%), *Campylobacter* spp. (2.25% vs. 1.34%), *Shigella* spp. (0.42% vs. 0.05%) whereas detection of *Salmonella* was slightly decreased (0.46% vs. 0.67%) when using the Seegene assay. An analysis of the time-to-result showed that the median dropped from 52.7 to 26.4 h when using the molecular panel testing. The Seegene Allplex™ GI-Bacteria(I) assay allows accelerated, reliable detection of major gastrointestinal bacteria roughly within 1 day. Workload is reduced, specifically in a low-prevalence setting.

## Introduction

Syndromic testing for infectious diseases, based on multiplexed molecular assays, has recently been introduced into clinical microbiology [1]. Amongst other diseases, gastrointestinal infections are addressed by a number of commercialized assays, some of them with an approval by the US food and drug administration [2]. Those assays detect multiple pathogens in parallel that can cause diarrhea. Routinely, diarrheal pathogens are identified by a combination of microscopy, antigen testing, culture, and singleplex PCR. The main disadvantage of the conventional approach is time-to-result, which for culture can take days, even for negative findings. Meanwhile multiple singleplex PCRs are available, but as clinical signs are often unspecific, ordering of multiple tests becomes uneconomic. Thus, syndromic, multiplex-based assays are promising to have clinical impact [3]. Advantages that are associated with syndromic, molecular testing include a rapid turnaround time that can affect clinical decision strategies including hospital admission, isolation, and infection control measures as well as sensitivities that often are superior to testing by culture [1]. Moreover, various pathogens can be detected in parallel, if clinical symptoms are unspecific. On the other hand, multiplex panels are expensive and clinical implementation strategies still have to be developed [4]. Panel compositions vary, but are mostly fixed; thus, laboratories will also have to cope with selecting panels that appropriately cover the local microorganisms. For diarrheal disease which is mostly self-limiting, routine testing for pathogens is not recommended but may be used in case of comorbidities, immunosuppression, bloody diarrhea, severe illness, decision of hospital admission, patients from community facilities, travelers, or patients with prolonged symptoms > 7 days [5, 6]. If available, an FDA-approved culture-independent method is recommended as an adjunct to conventional methods [5].

The Seegene Allplex real-time PCR assays allow simultaneous detection of up to seven pathogens within one reaction tube using a specific detection algorithm. Based on multiple quantification by real-time PCR and a specific interpretation software, for gastrointestinal infections, four panels, altogether comprising 25 pathogens, that can either be run in a combined or selected manner, are offered. Only few data are available for the Seegene Allplex Gastrointestinal assay so far [7, 8]: In comparison with Luminex xTAG GPP and BD MAX Enteric assays, the overall positive percentage agreements of Seegene, Luminex, and BD MAX were found to be 94%, 92%, and 78% [7]. No data on implementation of this assay in a routine workflow is available by now. We therefore report here on the implementation of the bacteria (I) panel, covering *Campylobacter* spp., *Clostridioides difficile* toxin B gene, *Salmonella* spp., *Shigella* spp.*/*EIEC, *Vibrio* spp., *Yersina enterocolitica*, and *Aeromonas* spp., instead of culture as a primary test for major bacterial diarrhea pathogens. Change of the diagnostic procedure was necessary to facilitate workflow, increase productivity, and reduce turnaround times for results, aims that have all been reported to be achievable by use of multiplex panel testing. A decision for the Seegene Allplex GB(I) assay was done based on cost estimates as the targets of the assay can be adopted to the local needs based on the use of selected panel tubes and as costs are lower than for fully automated, closed, but fixed systems.

## Materials and methods

### Multiplex PCR for syndromic panel diagnostic

Stool samples were analyzed using the Seegene Allplex™ GI-Bacteria (I) assay (Seegene, Seoul, South Korea) in combination with automated DNA extraction and PCR setup (Nimbus system) according to the manufacturer’s instructions. In brief, 150–200 μl fluid stool (equaling to 100–200 mg) were transferred from the stool container in 1 ml ASL buffer in a 2-ml tube (Qiagen, Hilden, Germany) using a flocked swab (PurFlock Ultra, Check Diagnostics, Germany) (Using a calibrated loop did not transfer enough material). The sample was vortexed, incubated for 10 min at room temperature, and then centrifuged for 2 min at 14,000 rpm. The 2-ml tube was directly used for DNA extraction. Alternatively, 800 μl supernatant was transferred into a new tube, if the ASL sample was very inhomogeneous after centrifugation. DNA extraction and PCR setup were done using STARMag Universal Cartridge kit (Seegene, Duesseldorf, Germany) in the Microlab Nimbus (Seegene) automated liquid handling workstation. The positive control was added after the automated PCR setup was manually done. The plate was removed from the Nimbus system, sealed with caps, and briefly centrifuged before analysis in a CFX96 cycler (Bio Rad, Germany). Results of the analysis were done using the Seegene Viewer software. Positive detection of *Salmonella* spp., *Yersina enterocolitica*, *Campylobacter* spp., *Shigella* spp.*/*EIEC, and *Vibrio* spp. was followed by an attempt to cultivate the respective pathogen from the original stool sample (“reflective culture”). Inhibited samples were diluted 1:3 in PBS before adding into ASL buffer and then repeated once.

### Detection by culture

Stool samples were analyzed according to routine procedures in the Institute of Medical Microbiology and Hygiene, Heidelberg, holding an accreditation according to DIN EN ISO 15189. In brief, stool samples were analyzed using blood (Becton Dickinson, Heidelberg, Germany), CIN, XLD agar, Campy (all bioMérieux, Marcy l’Étoile, France) selective agar, and a selenite broth (Becton Dickinson, Heidelberg, Germany), and were incubated for 24 and 48 h at 36 °C. Identification of suspicious colonies was done by MALDI-TOF (Bruker Daltonik, Bremen, Germany) using direct smear on target procedure. For further specification and confirmation, agglutination tests were performed for *Salmonella* spp., *Shigella* spp., and *Yersinia* spp. with specific antisera (SIFIN diagnostics, Berlin, Germany).

### *C. difficile* detection

*C. difficile* toxin B detection (from here on referred to as *C. difficile* detection) in routine clinical samples was done directly from stool samples using the molecular BD MAX C.diff assay according to the manufacturer’s instructions.

### Technical validation of the multiplex PCR assay’s performance

For determination of the limit of detection, 400 μl of a homogenous stool solution, tested negative for the respective pathogens, was spiked with 2 × 50μl of a 0.5 McF suspension of two pathogens each: *C. jejuni* (DSM4688), *S. cholerasuis* (ATCC554), *Y. enterocolitica* (ATCC9610), *S. flexneri* (ATCC29903), *C. difficile* (DSM27544), *Aeromonas hydrophila* (DSM30187), *Vibrio cholerae* (DSM100200). Thereafter, a 1:10 dilution series was produced (1 E7/ml to 1 E0/ml) and tested by the multiplex PCR as well as colony counting (for exact CFU determination) on the respective agar media. The test was repeated once with a 1:10 dilution series and then five times with a 1:3 dilution around the limit of detection. Moreover, 159 clinical stool samples (samples from the routine diagnostics in Heidelberg and samples from Lucerne) that had been analyzed before and that had been stored at − 80 °C for up 2 years were tested. For these samples, a culture result and/or a molecular result (BD MAX Enteric Panel, Biofire Filmarray, BD MAX C.diff) were available. In case of discrepant results, the samples were reanalyzed once by the same method. A composite of culture and/or molecular result was used as reference for the evaluation of the Seegene multiplex assay.

### Multiplex PCR implementation in routine diagnostics and clinical performance validation

The Seegene multiplex PCR was implemented as standard diagnostic procedure for requests of bacteria-induced diarrhea in 2017. Stool samples are analyzed once daily from Monday to Friday. At the weekends, stool samples are diluted in ASL buffer but stored until Monday (increased storage time was evaluated not to affect assay performance). At weekends, *C. difficile* detection was done using the BD MAX C.diff assay, whereas at weekdays, the *C. difficile* result as available from the Seegene multiplex PCR assay is reported. We did a comparison of the detection rates of the respective pathogens in a time period from November 6, 2017 to July 15, 2018, when using the Seegene multiplex PCR as the primary assay, followed by culture in case of positive detections, to the same time period 1 year before (November 6, 2017–July 15, 2017) when detection was done by culture only. Time-to-result was analyzed from laboratory information system (Swisslab, Nexus AG, Berlin) by using the entries “sample received” and “validation of final report”.

## Results

### Technical performance of the Seegene Allplex GI-B(I) multiplex PCR

We first determined the detection limit by spiking negative stool samples with a defined concentration of the included bacteria. It could be shown that the limit of detection (90% detection rate) was between 0.4 and 95.9 genome equivalents/PCR which is in the range reported by the manufacturer. Due to the dilution of the sample in the process of detection (1:10 for preparation in ASL buffer, 5 μl/100 μl of the nucleic acid eluate within the PCR reaction), the assay detected in detail: 1.4 E5 CFU/ml for *Campylobacter* spp., 3.7 E4 CFU/ml *Salmonella* spp., 9.5 E2 CFU/ml for *Shigella* spp., 1.4 E4 CFU/ml for *Yersina enterocolitica*, and 5.7 E3 CFU/ml for *Aeromonas* spp. Next, we tested the assay with *N* = 159 clinical samples for which a positive result of the included bacteria had been obtained earlier. A compound reference of culture and/or positivity by a molecular assay was used as standard. The sensitivity of the assay was 100% for *Campylobacter* spp., 92% for *Salmonella* spp., and 89% for *Aeromonas* spp. For *Yersinia enterocolitica*, the sample numbers were small: 3 samples, positive by culture were detected by the multiplex PCR, whereas 4 samples for which only a molecular assay was positive were negative with the Seegene multiplex PCR. For *Shigella* spp., detection sensitivity was 100% when compared with culture but 83% when another molecular test was added. Sensitivity for *C. difficile* detection was evaluated by a comparison with the BD MAX C.diff kit, a singleplex PCR, and was 93.9%.

### Routine results with a multiplex PCR workflow

**Fig. 1:**
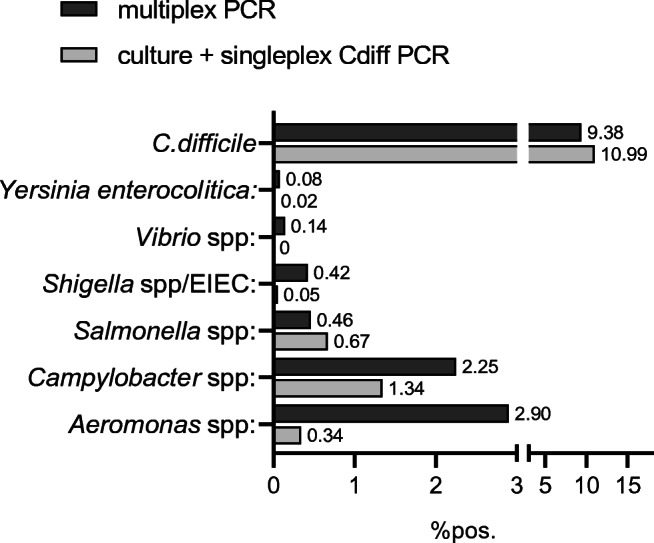
Performance of the Seegene Allplex™ GI-B(I) multiplex assay in a routine setting based on a before-after comparison approach. Clinical samples obtained by multiplex PCR analysis during November 2017–June 2018 (*N* = 5032) were compared with culture results from the corresponding previous time period November 2016–June 2017 (*N* = 4173). *C. difficile* toxin B detection was compared with a singleplex PCR

The Seegene multiplex PCR was introduced into the routine workflow as the standard diagnostic procedure replacing primary bacterial culture. Stool samples were analyzed as a batch (20–50 samples) once daily at weekdays; no diagnostic was offered at weekends. *C. difficile* detection was done using the Seegene multiplex PCR at weekdays, but by singleplex BD MAX PCR at weekends. The workflow allowed a considerable decrease in hands-on times as preparation and setup of the test was largely automated (estimate 3 h/day for panel diagnostic vs. 1 FTE for culture handling). Within a low-prevalence setting, the workflow resulted in only few cultures that were needed to be set up as confirmation for samples positive by the molecular assay. To evaluate whether molecular detection also increased detection rates of classical bacterial diarrhea pathogens, we compared results from an 8-month time period using the molecular assay with a similar period in the year before using culture. Of note, within this time period, the reported German-wide detection rates for the included pathogens (data from the national health authorities) were comparable. With the introduction, we observed increased detection rates for *Aeromonas* spp. (8.5-fold increase, 2.90% vs. 0.34%), *Campylobacter* spp. (1.7-fold increase, 2.25% vs. 1.34%), and *Shigella* spp. (8.4-fold increase, 0.42% vs. 0.05%) whereas detection of Salmonella was slightly decreased (0.46% vs. 0.67%) (Fig. 1, Table 1). *C. difficile* toxin B detection was 9.38% vs 10.99% by the singleplex BD MAX PCR. Upon reflex testing by culture, 56% and 61% of the positive results for *Campylobacter* spp. and *Salmonella* spp. could be confirmed (Table 1). For samples with a positive result for *Shigella* spp./EIEC, culture for *Shigella* spp. was only positive in 14% of the samples that gave a positive molecular signal. The Seegene assay uses a shared target gene for *Shigella* spp. and EIEC, but only for *Shigella* a specific culture method exists. This might contribute to the observed discrepancies. Of note, all of the tested patients had clinical symptoms of diarrhea. All positive results for *Y. enterocolitica* were confirmed by culture. The 7 samples positive for *Vibrio* were from two travelers with culture-confirmed *V. cholera* infection.

**Table 1:** Reflex culture of samples positive for any analyte in the Seegene Allplex™ GI-B(I) multiplex assay

	Positive (*N*)	Confirmation by culture
*Aeromonas* spp.	146	n.d.
*Campylobacter* spp.	113	63 (56%)
*Salmonella* spp.	23	14 (61%)
*Shigella* spp./EIEC	21	3 (14%)
*Vibrio* spp.	7	4 (57%)
*Y. enterocolitica*	4	4 (100%)
*C. difficile* toxin B	422	n.d.

*n.d.*, not done

### Reduced time-to-result with a multiplex PCR diagnostic algorithm

**Fig. 2:**
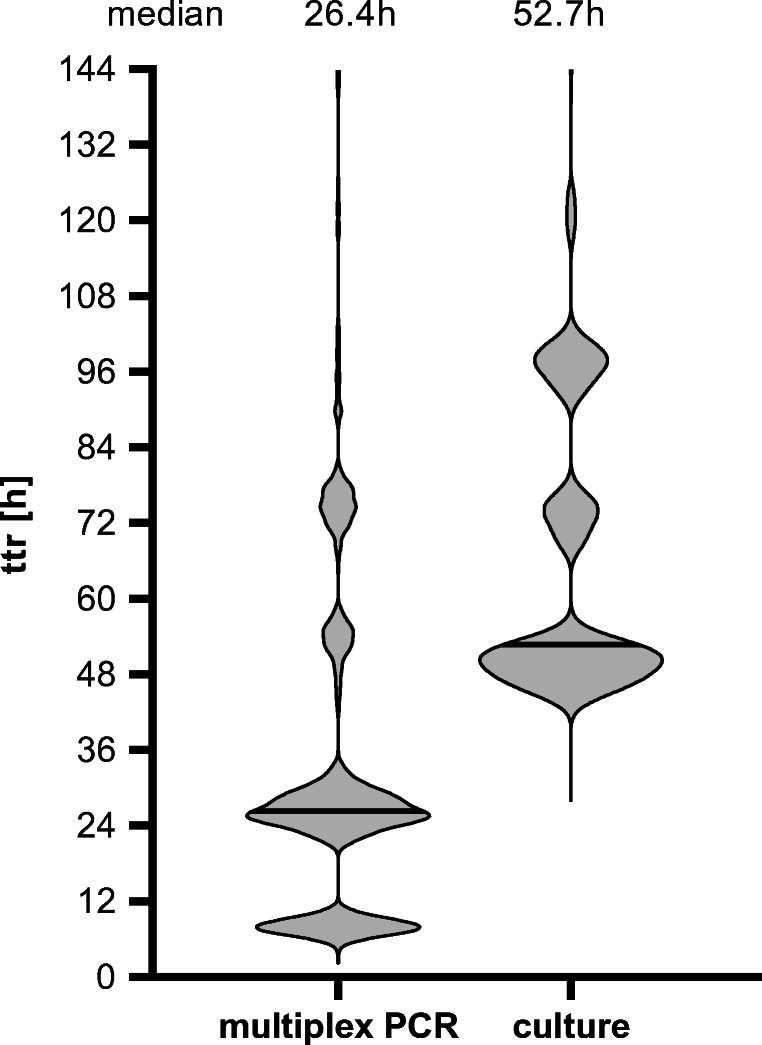
Time to final result (including reflex testing) for the data of Fig. 1. Multiplex PCR, Seegene Allplex™ GI-B(I) multiplex assay, vs. culture

With the newly implemented molecular diagnostic algorithm, time to final result decreased from 52.7 h (median) by culture to 26.4 h by multiplex PCR (including secondary culture if necessary) (Fig. 2). The differences were significant (Kruskal-Wallis test *p* < 0.0001). Thus, results were available roughly 1 day earlier. Results of the PCR were obtained in 23.9% of the samples even at the same day (< 14 h) and for 74.0% of the samples within the next day (< 38 h). Of note, in the current workflow, no diagnostic by multiplex PCR was done at weekends.

## Discussion

We report on the implementation of the molecular Seegene Allplex™ GI-Bacteria(I) assay for primary detection of bacterial diarrheal pathogens within a low-incidence setting. The assay reliably detected major gastrointestinal bacteria including *Campylobacter*, *Salmonella*, *Shigella/*EIEC, and *Yersinia* which are to be notified to German health authorities. By performing a before-after comparison, we observed that clinical detection rates for *Campylobacter*, *Shigella*, and *Aeromonas* increased after introduction of the molecular panel workflow. Although a direct side-by-side comparison could not be done, the use of an equivalent time period and the data from the German health reporting system showing comparable German-wide epidemiology for the chosen periods justify such kind of comparison. Findings are in line with reports from other multiplex PCRs that show increased sensitivity for detection of gastrointestinal pathogens by molecular methods as compared with culture [9]. For the Filmarray GI Panel, a multicenter study showed detection of at least one pathogen in 54.2% of the samples vs. 18.1% with conventional techniques [10]. For the Luminex Gastrointestinal Pathogen Panel, 22.1% vs. 12% was reported [2], and other studies confirmed these differences [11, 12]. In contrast, detection of *Salmonella* by molecular panel was slightly less sensitive, and differences probably would have been even bigger if an enrichment broth was used. On the other hand, the Seegene assay also identified samples positive for *Salmonella*, for which culture was negative, indicating that both methods may find additional positive samples. Indeed, another study using the Luminex GPP assay also showed a positive percent agreement between culture and multiplex PCR for *Salmonella* of only 78.2% [13], with additional positive samples by both methods. Shortcomings in accuracy of molecular detection for *Salmonella* and *Yersinia* have also been reported by others [12, 14, 15]. In a comparative study with Biofire-, Luminex-, and Verigene-panels, two of the three assays had a sensitivity < 85% for detection of *Salmonella* whereas the other bacterial pathogens were detected very well [16]. Caution might be indicated for the use of multiplex PCR testing when specifically *Salmonella* shall be detected as for example in food industry workers.

Upon positive molecular detection we tried to cultivate the respective bacteria, a strategy of reflective culture. Thus, only a very limited number of samples had to undergo the time-consuming and laborious process of cultivating. For *Salmonella* and *Campylobacter*, this was only successful in 56% and 61% of the samples. As the patients suffered from diarrhea, a correct molecular diagnosis was assumed, but formally, it cannot be excluded that false-positive signals were included. Thus, it remains important that interpretation of results of multiplex panels is always done considering the patient’s symptoms, history, and risk profile [17]. Probably, not a single “gold standard” for diagnostics exists.

The workload of the molecular Seegene Allplex GI-Bacteria (I) assay was reduced as compared with the culture process which has also been reported in other studies (reviewed in [1]). One of the most important findings was the significantly reduced time-to-result with the implementation of a molecular multiplex assay. We observed a reduction of more than 1 day, and in nearly a quarter of all samples, a same-day result was achieved. Of note, various molecular panels offer a rapid detection, yet time-to-result also depends on the strategy of implementation. Here, in a routine setting, a significant reduction could be obtained. Similar savings were observed for the Biofire FilmArray with a reduction from 47 to 18 h [18]. For the Luminex assay, implemented in a routine setting, turnaround times were reduced from 66.5 to 41.8 h in one study [2], where it was noted that conventional testing for *C. difficile* was faster (17.3 h). In our implementation strategy, *C. difficile* result was obtained at latest the next day after sample delivery. Yet, as we did not offer multiplex testing at weekends, we still had to run a singleplex PCR for those indications.

As sensitivity of molecular detection is high, within a low-prevalence setting, the negative predictive value will be high, thus allowing a more rapid decision on the necessities of isolation and hygiene measurements. Based on the specific implementation, the time-to-result might be decreased further, but even with a batched protocol once a day, we had a considerable reduction. This is in line with reports for other diarrheal panels.

Implementation of a molecular assay has also to be considered under economical viewpoints. In our specific case, the change resulted in only moderately increased direct laboratory costs, because the previous procedure already involved culture plus a singleplex PCR for *C. difficile*, with the latter being responsible for the majority of the costs. A cost estimate for consumables was 75 K € in the 8-month period 2016–2017 (11 K € and 64 K € for culture and *C. difficile* detection with 4173 and 4141 samples, respectively, many of them with both requests) and 86 K € for molecular detection (71 K € and 14 K € for 5032 samples by multiplex PCR and 938 samples with singleplex *C. difficile* PCR). Of note, this estimate does neither consider any laboratory savings by the reduced workload nor any clinical savings. It was cost-effective, because a PCR for *C. difficile* could be replaced in parallel. Of course, additional economical beneficial effects come from increased diagnosis of defined pathogens and savings in isolation procedures that have not been calculated in this study. In a cost-benefit analysis for Luminex GPP testing against conventional assays, laboratory costs for 800 patients in an 8-month period increased by £22,283 but resulted in savings of £66,765, mostly due to reduction in isolation time [4]. The authors concluded that specifically, a rapid negative result could reduce cost by decreasing isolation times. Studies with gastrointestinal panels were shown to improve patient care by rapid identification of pathogens, reduction of numbers of additional test, less endoscopy and abdominal radiology, less prescription of antibiotics, and earlier release from hospital [3, 19].

Economical implementation of syndromic panel testing might also involve stratification for the use of such assay: It was shown that panel testing in patients developing diarrhea more than 3 days after hospital admission has only low yield and thus could be excluded in a decision algorithm [20].

Taken together, the Seegene Allplex™ GI-Bacteria(I) assay allowed an accelerated, reliable detection of major gastrointestinal bacteria, a reduction in the workload, and a significant decrease in the time-to-result. It may be implemented in a routine workflow as primary assay for the detection of bacteria-induced diarrhea and has advantages in a setting with high negative detection rate.
